# Case Report: Isolated, unilateral oculomotor palsy with anti-GQ1b antibody following COVID-19 vaccination

**DOI:** 10.12688/f1000research.74299.1

**Published:** 2021-11-11

**Authors:** Takafumi Kubota, Takafumi Hasegawa, Kensuke Ikeda, Masashi Aoki

**Affiliations:** 1 Department of Neurology, Tohoku University Graduate School of Medicine, Sendai, Miyagi, 980-8574, Japan

**Keywords:** oculomotor nerve palsy, Miller Fisher syndrome, anit-GQ1b antibody, ganglioside, COVID-19, vaccination, IVIG

## Abstract

Neurological complications following vaccinations are extremely rare, but cannot be eliminated. Here, we report the first case of unilateral oculomotor nerve palsy (ONP) with anti-GQ1b antibody after receiving the Pfizer-BioNTech COVID-19 (BNT162b2) mRNA vaccine.

A 65-year-old man developed diplopia and ptosis in the right eye 17 days after vaccination, without preceding infection. Neurological examination revealed mild blepharoptosis, limitation of adduction, and vertical gaze on the right side. Increased levels of anti-GQ1b ganglioside antibody in the serum and albuminocytologic dissociation in the cerebrospinal fluid were detected. Cranial magnetic resonance imaging showed swelling and enhancement of the right oculomotor nerve. The patient was diagnosed with right ONP accompanied with anti-GQ1b antibody, and intravenous immunoglobulin (IVIG) therapy for 5 days was administered. The limitation of adduction and vertical gaze improved, and ptosis markedly resolved after IVIG treatment. Given the temporal sequence of disease progression, laboratory findings, and a favorable response to IVIG, a causal relationship cannot be ruled out between the occurrence of ONP and COVID-19 immunization. Since immunomodulatory treatments significantly hasten the recovery and minimize the residual symptoms in anti-GQ1b antibody syndrome, clinicians should be aware of this clinical condition following COVID-19 vaccination.

## Introduction

Oculomotor nerve palsy (ONP) is a neurological condition that manifests as diplopia, ptosis, and pupillary mydriasis. The various etiologies of ONP include cerebrovascular disease, cerebral aneurysm, diabetes, tumor, infection, collagen disease, hyperthyroidism, and Tolosa-Hunt syndrome ^1^. In some cases, ONP can be caused by an aberrant immune response that develops directly against ganglioside GQ1b, a sialic acid-containing glycosphingolipid enriched in the paranodal region in the III (oculomotor), IV (trochlear), and VI (abducens) cranial nerves ^2^. The para-infectious, immune-mediated ONP, along with ataxia and loss of tendon jerks, was originally described by Charles Miller Fisher as a variant of Guillain-Barré Syndrome (GBS) ^3^. Since there are incomplete or atypical forms of Miller Fisher syndrome (MFS), an umbrella term, “anti-GQ1b antibody syndrome” has emerged to encompass these clinical conditions ^4^.

In addition to an antecedent infectious illness, vaccine-mediated immunization can trigger GBS and MFS ^5– 8^, for example, MFS following influenza ^6– 8^, pneumovax ^8^, and DPT (diphtheria, pertussis, tetanus toxoid) vaccination ^5^ has been reported. GBS has been listed as a very rare neurological complication of the COVID-19 vaccine ^9– 15^. However, to the best of our knowledge, there have been no case reports of isolated, unilateral ONP with anti-GQ1b antibody following vaccination. Here, we report an adult case of acute-onset right ONP with anti-GQ1b antibody following COVID-19 vaccination with a literature review.

## Case description

A 65-year-old Asian male office worker began to notice persistent double vision without preceding upper respiratory or gastrointestinal infection. The diplopia worsened in the left gaze, and three days later, he developed right ptosis. He was vaccinated with a second dose of Pfizer-BioNTech COVID-19 (BNT162b2) mRNA vaccine 17 days before his presentation. His medical history included a seven-year history of diabetes, glaucoma, and benign paroxysmal positional vertigo. He did not have diabetic retinopathy or neuropathy in his right eye. His medication included one tablet per day of Canalia ^® ^(teneligliptin and canagliflozin), a diabetic combination drug which the patient had been taking for one year and one drop per day of prostaglandin analogue eye drops for glaucoma (time taken for unknown). 

**Figure 1.**
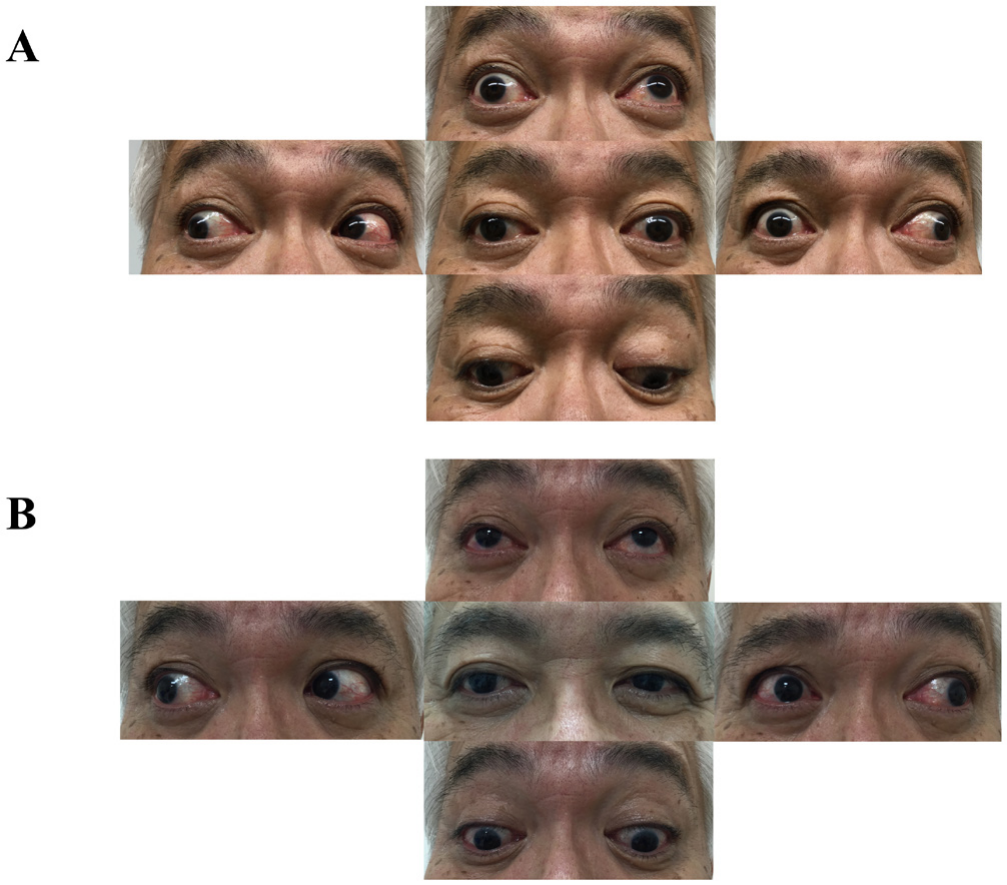
Eye movement of the patient demonstrating right oculomotor nerve palsy. ( **A**) Mild blepharoptosis, limitation of adduction and vertical gaze on the right side on day 30. ( **B**) The limitation of adduction and vertical gaze improved and ptosis completely resolved after IVIG treatment (day 52).

The general medical condition of the patient on admission (day 22) was unremarkable. Neurological examination revealed mild blepharoptosis, limitation of adduction, and vertical gaze on the right side ( Figure 1A) with convergence insufficiency. Pupils were slightly asymmetric (right: 3.5 mm, left: 3.0 mm) and the right pupil was slightly slowly reactive to light. The other cranial nerves were preserved normally. These findings were consistent with the diagnosis of right ONP. Gait was normal, with no evidence of muscle weakness, ataxia, or sensory disturbances. Deep-tendon reflexes are normally elicitable.

### Diagnostic assessment, therapeutic intervention, follow-up, and outcomes

**Figure 2.**
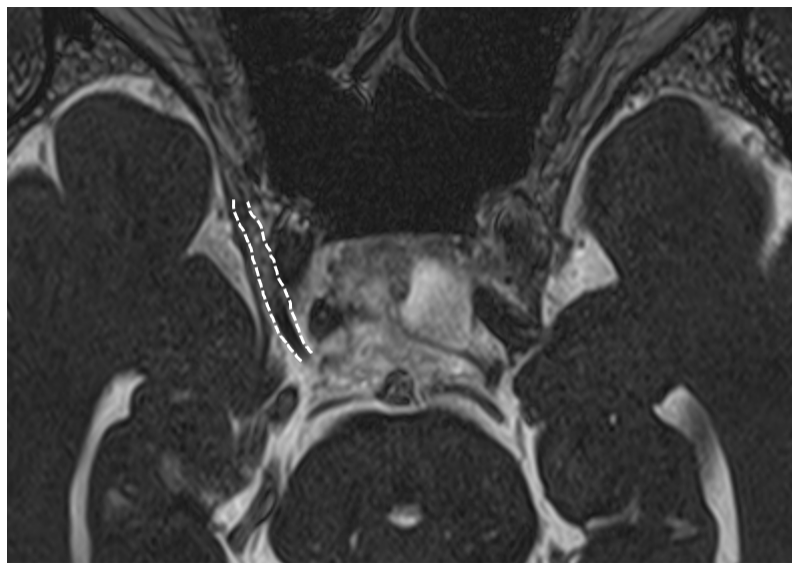
Contrast-enhanced CISS-MRI demonstrates the swelling and enhancement in the right oculomotor nerve (indicated by *white dotted lines*).

Routine hematological and biochemical analyses, including thyroid function, were normal except for the elevation in glucose concentration 162 mg/dL (normal range: 78–109 mg/dL) and HbA1c level 7.8% (normal range: 4.6–6.2%). Serological tests identified the presence of anti-GQ1b IgG antibody (1.82, normal cut-off index <1), a pathognomonic marker for MFS. Other antibodies against glycoconjugates, including ganglioside GM1, antinuclear antibodies, perinuclear antineutrophil cytoplasmic antibody (ANCA), cytoplasmic ANCA, and acetylcholine receptor antibodies were negative. Cerebrospinal fluid showed mild albuminocytologic dissociation with protein levels of 52 mg/dL (normal range: 10–40 mg/dL) and 2 mononuclear cells/mm ^3 ^(normal range: 0–5 cells/mm ^3^). High-resolution, constructive interference in steady-state magnetic resonance imaging (CISS-MRI) showed swelling with gadolinium enhancement in the right cavernous segment of the oculomotor nerve ( Figure 2), but no signs of aneurysm, tumor, and inflammation in the cavernous sinus and orbital apex were noted. A nerve conduction study in the limbs was normal.

Based on these clinical and laboratory findings, we diagnosed the patient with isolated, unilateral ONP associated with anti-GQ1b antibody and administered intravenous immunoglobulin (IVIG, 400 mg/kg) for consecutive 5 days. On the fourth day of IVIG administration (day 36), the limitation of the vertical gaze and ptosis mildly improved. There were no adverse events during or after the IVIG treatment. The patient was discharged on day 40 and was followed up at an outpatient clinic on day 52. The limitation of the adduction and vertical gaze markedly improved and ptosis completely resolved on day 52 ( Figure 1B). The patient also noticed an improvement in his diplopia. The patient was afraid of receiving further vaccinations, including the COVID-19 vaccine. We explained to him that the incidence of GBS and MFS caused by vaccinations are extremely rare and the causal link between neurological complication and COVID-19 vaccinations is still unclear. It is undoubtedly true that benefits of vaccination outweigh the risks and the truth of the matter is that rare side effects shouldn't rule out vaccines. If he is going to be vaccinated in the future, we will carefully watch his condition and seek medical attention as soon as possible if he experiences any complications.

## Discussion

We report the first case of isolated unilateral ONP with anti-GQ1b antibody following COVID-19 vaccination. Clinically, there were many similarities between our case and the previous four cases of unilateral ONP with anti-GQ1b antibody ( Table 1) ^16– 18^. First, all cases, including ours, had ptosis without ataxia, and three cases showed normal deep tendon reflexes. Second, three patients demonstrated albuminocytologic dissociation in the CSF. Third, all of the cases showed a normal pattern in the nerve conduction studies. Finally, four patients were successfully treated with IVIG and/or steroids, with different recovery periods ranging from 22 days to 6 months ^16– 18^. From an etiopathological point of view, two cases of isolated ONP with anti-GQ1b antibody were preceded by acute upper respiratory tract infection or gastroenteritis within two weeks of onset ^17, 18^. On the other hand, there have been no case reports of vaccine-induced, isolated ONP with anti-GQ1b antibody. Similar to GBS, the majority of MFS and other anti-GQ1b antibody-associated disorders showed a good response to immunotherapy, such as IVIG and plasmapheresis. However, if ONP is the sole manifestation of anti-GQ1b antibody syndrome, it can be difficult to diagnose, leading to a substantial therapeutic delay. In agreement with our case, cranial MRI demonstrated abnormal swelling, T2 hyperintensity, and gadolinium enhancement of the affected oculomotor nerve in classic forms of MFS, providing a useful tool for early diagnosis ^19– 21^.

**Table 1.** Summary of previous reports and present case.

**Author and year**	Lee 2008	Lee 2008	Ichikawa 2002	Ueno 2017	Present case
**Age (year)**	27	30	47	68	65
**Sex**	Female	Male	Male	Male	Male
**Preceding vaccination**	(-)	(-)	(-)	(-)	COVID-19 (Pfizer)
**Preceding infection**	Gastroenteritis	URI	URI	Gastroenteritis	(-)
**Time between preceding event and onset**	NA	NA	14 days	8 days	17 days
**Affected eye side**	Right	Right	Left	Right	Right
**Ptosis**	(-)	(+)	(+)	(+)	(+)
**Gage limitation**	Vertical	Adduction and vertical	Adduction and vertical	Adduction and vertical	Adduction and vertical
**Ataxia**	(-)	(-)	(-)	(-)	(-)
**Deep tendon reflex**	Normal	Decreased	Normal	Decreased	Normal
**CSF**	Normal	Albuminocytologic dissociation	Albuminocytologic dissociation	Normal	Albuminocytologic dissociation
**NCS**	Normal	Normal	Normal	Normal	Normal
**Contrast-enhanced MRI**	NA	NA	NA	NA	Enhancement in oculomotor nerve
**Treatment**	IVIG or Steroid	IVIG or Steroid	IVIG and Steroid	No	IVIG
**Recovery period**	Follow up loss	6 months	28 days	44 days	36 days (mild improvement)

URI, upper respiratory infection; CSF, cerebrospinal fluid; NCS, Nerve conduction study; MRI, magnetic resonance imaging; NA, Not available; IVIG, intravenous immunoglobulin.

A growing concern among recent coronavirus vaccines is vaccine-related side effects. The most commonly observed adverse events with COVID-19 vaccines are fatigue, headache, muscle and joint pain, fever, pain at the site of injection, and to a much lesser degree, severe allergic reactions including anaphylaxis. The occurrence of these side effects can be predicted based on what is already known about the clinical trials of other vaccines. While not all reported side effects are directly related to vaccine administration, life-threatening side effects such as thromboembolism and neurological complications, including GBS, have also been reported following COVID-19 vaccines. In the United States, as of July 13, 2021 there were 100 preliminary reports of GBS after receiving the Janssen COVID-19 vaccine and 1 death after 12.5 million vaccine doses administered. GBS usually develops 3–22 days after the administration of COVID-19 vaccines ^9– 15^. Although MFS following COVID-19 vaccination has not been reported so far, MFS can be observed from 5 to 21 days after immunization with influenza ^6– 8^, pneumovax ^8^, and DPT vaccines ^5^. Similarly, our case also presented with unilateral ONP with elevated anti-GQ1b antibody 17 days after Pfizer-BioNTech COVID-19 (BNT162b2) vaccination without any preceding infection. Based on the temporal sequence of disease progression, laboratory findings, and a favorable response to immunotherapy, the possibility that preceding COVID-19 vaccination may provoke unfavorable immune responses, leading to ONP in our patient, cannot be ruled out.

It should be noted that ONP is the most common cranial neuropathy in patients with diabetes ^22^. Diabetes not only causes ischemic neuropathy, but also induces chronic low-level inflammation in peripheral nerves through the elevation of various inflammatory markers such as C-reactive protein, tumor necrosis factor, and interleukin-6 ^22^. Thus, one can imagine that the pre-existing diabetes in our case might impair oculomotor nerve function, thereby aggravating demyelinating oculomotor damage by the anti-GQ1b antibody. Indeed, diabetes has been reported as a risk factor for the exacerbation and poor outcomes of GBS ^23– 25^.

## Conclusion

Unilateral, isolated ONP with anti-GQ1b antibody was observed following COVID-19 vaccination. Insufficient recognition of this treatable condition often leads to misdiagnosis, which delays the receipt of adequate immunomodulatory therapy. Physicians should consider this rare clinical entity, even when the classical triad of MFS is absent. While the benefits of COVID-19 vaccination substantially outweigh the rare, possible adverse events, healthcare professionals should carefully monitor the hazardous effects of all COVID-19 vaccines and continue to work closely to manage potential risks and to harness science and big data to drive feedback and recommendations.

## Consent

Written informed consent was obtained from the patient for the publication of this case report and any associated images.

## Data availability

All data underlying the results are available as part of the article and no additional source data are required.
