# Supplementary material for: Case Report: Isolated, unilateral oculomotor palsy with anti-GQ1b antibody following COVID-19 vaccination
Source: F1000Res. 2022 Apr 5;10:1142. Originally published 2021 Nov 11. [Version 2] doi: 10.12688/f1000research.74299.2 (PMC8990240; doi:10.12688/f1000research.74299.2)
Supplement: Peer review history

# Peer review: Reviewer response for version 2

**DOI:** 10.5256/f1000research.123036.r129879

**Published:** 2022-04-19

**Authors:** Chieko Suzuki

**Affiliations:** 1 Department of Neurology, Graduate School of Medicine, Hirosaki University, Hirosaki, Japan

The manuscript has been revised well. I have no further comments.

Are enough details provided of any physical examination and diagnostic tests, treatment given and outcomes?

Yes

Is the case presented with sufficient detail to be useful for other practitioners?

Yes

Is sufficient discussion included of the importance of the findings and their relevance to future understanding of disease processes, diagnosis or treatment?

Yes

Is the background of the case’s history and progression described in sufficient detail?

Yes

Reviewer Expertise:

Neuropathy

I confirm that I have read this submission and believe that I have an appropriate level of expertise to confirm that it is of an acceptable scientific standard.

# Peer review: Reviewer response for version 2

**DOI:** 10.5256/f1000research.123036.r129880

**Published:** 2022-04-07

**Authors:** Miguel García-Grimshaw

**Affiliations:** 1 Department of Neurology and Psychiatry, Instituto Nacional de Ciencias Médicas y Nutrición Salvador Zubirán, Mexico City, Mexico; 2 Department of Neurology, Hospital General Tijuana, Tijuana, Mexico

I have no further comments. The author complied with the reviews of my first revision.

Are enough details provided of any physical examination and diagnostic tests, treatment given and outcomes?

Partly

Is the case presented with sufficient detail to be useful for other practitioners?

Partly

Is sufficient discussion included of the importance of the findings and their relevance to future understanding of disease processes, diagnosis or treatment?

Partly

Is the background of the case’s history and progression described in sufficient detail?

Yes

Reviewer Expertise:

Currently working on acute and long-term neurologic manifestations of COVID-19 and neurologic adverse events following immunization against SARS-CoV-2.

I confirm that I have read this submission and believe that I have an appropriate level of expertise to confirm that it is of an acceptable scientific standard.

# Peer review: Reviewer response for version 1

**DOI:** 10.5256/f1000research.78037.r126605

**Published:** 2022-03-22

**Authors:** Miguel García-Grimshaw

**Affiliations:** 1 Department of Neurology and Psychiatry, Instituto Nacional de Ciencias Médicas y Nutrición Salvador Zubirán, Mexico City, Mexico; 2 Department of Neurology, Hospital General Tijuana, Tijuana, Mexico

In this report, the authors describe the case of a male who developed an isolated unilateral oculomotor palsy following immunization with BNT162b2 and positivity anti-GQ1b antibody.

 Real-world evidence (outside clinical trials) is essential to detect potential adverse events of these newly developed vaccines. However, this case does not fulfill the clinical diagnostic criteria for the Miller Fisher syndrome (MFS) variant of Guillain-Barré syndrome (GBS). 

 It would be interesting if the author could discuss the positivity of anti-GQ1b antibody in the general population patients (without clinical suspicion of GBS), including sensitivity and specificity of this antibody for diagnosing MFS.

 In the discussion section, the author describes the possibility of diabetic cranial neuropathy. It would be interesting to know if the patient was tested for oligoclonal bands in the cerebrospinal fluid and serum anti-AQP-4 antibodies as part of unilateral optic neuritis differential diagnosis as his contrast-enhanced MRI demonstrates swelling and enhancement in the right oculomotor nerve. 

 Lastly, it would be interesting if the author could discuss or speculate on some of the pathophysiological mechanisms on how mRNA-based vaccines may be related to GBS or MFS.

Are enough details provided of any physical examination and diagnostic tests, treatment given and outcomes?

Partly

Is the case presented with sufficient detail to be useful for other practitioners?

Partly

Is sufficient discussion included of the importance of the findings and their relevance to future understanding of disease processes, diagnosis or treatment?

Partly

Is the background of the case’s history and progression described in sufficient detail?

Yes

Reviewer Expertise:

Currently working on acute and long-term neurologic manifestations of COVID-19 and neurologic adverse events following immunization against SARS-CoV-2.

I confirm that I have read this submission and believe that I have an appropriate level of expertise to confirm that it is of an acceptable scientific standard, however I have significant reservations, as outlined above.

# Response

**Published:** 2022-03-30

**Authors:** Takafumi Hasegawa

**Affiliations:** Tohoku University, Japan

Comment #1: It would be interesting if the author could discuss the positivity of anti-GQ1b antibody in the general population patients (without clinical suspicion of GBS), including sensitivity and specificity of this antibody for diagnosing MFS.

**Reply: **Thank you very much for your valuable comment. From two previous studies, all ten patients with MFS were positive for anti-GQ1b antibody, whereas all 81 control people without GBS were negative. Thus, the sensitivity and specificity of anti-GQ1b antibody in the patients with MFS should be very close to 100% (Chiba A et al. Ann Neurol (1992) 31:677–679. Willison HJ et al. J Neurol Neurosurg Psychiatry (1993) 56:204–206). We added this point in the first paragraph of introduction as follows: “Compared to control subjects without neurological complications, the sensitivity and specificity of anti-GQ1b antibody in the patients with MFS are very close to 100% (2,4).Since there are incomplete or atypical forms of Miller Fisher syndrome (MFS), an umbrella term, “anti-GQ1b antibody syndrome” has emerged to encompass these clinical conditions (5).”

 Comment #2: In the discussion section, the author describes the possibility of diabetic cranial neuropathy. It would be interesting to know if the patient was tested for oligoclonal bands in the cerebrospinal fluid and serum anti-AQP-4 antibodies as part of unilateral optic neuritis differential diagnosis as his contrast-enhanced MRI demonstrates swelling and enhancement in the right oculomotor nerve.

**Reply: **We appreciate this reviewer’s constructive comment. Oligoclonal bands were negative in this case, but we did not measure anti-AQP-4 antibodies. It is true that anti-AQP-4 antibodies can cause oculomotor nerve palsy in a very rare case (Yasuda K et al. J Clin Neurosci. 2019 Aug;66:271-272.); however, our case did not have any other symptoms and signs of NMOSD. Hence, we think it unlikely that anti-AQP-4 antibodies contribute to the manifestation of oculomotor nerve palsy in our case. Taking the above into consideration, we added the data of oligoclonal bands and myelin basic protein in the diagnostic assessment section as follows: “Oligoclonal bands were negative and myelin basic protein was less than 31.3 pg/mL (normal range: < 102 pg/mL).”.

 Comment #3: Lastly, it would be interesting if the author could discuss or speculate on some of the pathophysiological mechanisms on how mRNA-based vaccines may be related to GBS or MFS.

**Reply: **Thank you very much for your comment. We add the possible mechanism by which mRNA-based vaccine produce anti-GQ1b antibody and subsequent neurological deficits in the second paragraph of the discussion as follows: “BNT162b2 vaccine of messenger RNA enter the body and export spike proteins on the cell, which provoke the production of antibodies and T cell reactions (19). These immunological alterations may produce neutralizing antibodies as well as anti-GQ1b antibody, thereby leading to unfavorable neurological complications.”

# Peer review: Reviewer response for version 1

**DOI:** 10.5256/f1000research.78037.r99811

**Published:** 2021-11-30

**Authors:** Chieko Suzuki

**Affiliations:** 1 Department of Neurology, Graduate School of Medicine, Hirosaki University, Hirosaki, Japan

This report shows unilateral oculomotor nerve palsy associated with anti- GQ1b antibody following COVID-19 vaccination. It is important because there is a growing concern about the adverse effects of COVID-19 vaccines worldwide.

 Anti GQ1b antibody-associated isolated oculomotor nerve palsy is rare. On the other hand, diabetic oculomotor nerve palsy is a common disease. It is necessary to examine whether diabetes has any effect on this condition. In this case, treatment was started more than one month after the onset of the disease.

 If the condition is related to GQ1b, recovery phase may begin. Were there any signs of recovery before starting treatment?

1. Elevated CSF protein is often observed in diabetes mellitus. Do you have follow-up data of CSF? If this condition is associated with GQ1b, there may be changes in CSF protein.
2. Contrast-enhanced findings of the oculomotor nerve on MRI have been reported in diabetic oculomotor palsy and idiopathic oculomotor palsy (Zhao *et al.* 2021 ^1^; Yang *et al.* 2020 ^2^). This finding is not specific to MFS.

Are enough details provided of any physical examination and diagnostic tests, treatment given and outcomes?

Yes

Is the case presented with sufficient detail to be useful for other practitioners?

Yes

Is sufficient discussion included of the importance of the findings and their relevance to future understanding of disease processes, diagnosis or treatment?

Yes

Is the background of the case’s history and progression described in sufficient detail?

Yes

Reviewer Expertise:

Neuropathy

I confirm that I have read this submission and believe that I have an appropriate level of expertise to confirm that it is of an acceptable scientific standard.

## References

[1] : Diabetic Oculomotor Nerve Palsy Displaying Enhancement of the Oculomotor Nerve in the Orbit and Cavernous Sinus on MRI. *Eur Neurol* .2021;84(4) : 10.1159/000514100 246-253 10.1159/000514100 33975309

[2] : Clinical Significance of MRI Contrast Enhancement of the Oculomotor Nerve in Ischemic Isolated Oculomotor Nerve Palsy. *J Clin Neurol* .2020;16(4) : 10.3988/jcn.2020.16.4.653 653-658 10.3988/jcn.2020.16.4.653 33029972PMC7541971

# Response

**Published:** 2022-03-30

**Authors:** Takafumi Hasegawa

**Affiliations:** Tohoku University, Japan

Comment #1: If the condition is related to GQ1b, recovery phase may begin. Were there any signs of recovery before starting treatment?

**Reply: **Thank you very much for your comment. There were no obvious signs of recovery before IVIG treatment. He had gradually improved after the therapy. To further clarify the process of recovery, we added the last follow-up when he completely recovered on day 77 in the second paragraph of the diagnostic assessment section as follows: “He completely recovered on day 71 ( **Figure 1 C**).”

 Comment #2: Elevated CSF protein is often observed in diabetes mellitus. Do you have follow-up data of CSF? If this condition is associated with GQ1b, there may be changes in CSF protein.

**Reply: **Thank you very much for your comment. We are sorry but we did not perform follow-up CSF analysis. As you mentioned, albuminocytologic dissociation in CSF might have improved after the therapy.

 Comment #3: Contrast-enhanced findings of the oculomotor nerve on MRI have been reported in diabetic oculomotor palsy and idiopathic oculomotor palsy (Zhao et al. 20211; Yang et al. 20202). This finding is not specific to MFS.

**Reply: **We humbly accept this reviewer’s comment. We deleted the following sentence in the 1 ^st^ paragraph of the discussion: “In agreement with our case, cranial MRI demonstrated abnormal swelling, T2 hyperintensity, and gadolinium enhancement of the affected oculomotor nerve in classic forms of MFS, providing a useful tool for early diagnosis.”
